# Supplementary figures and images for: Solving the stochastic dynamics of population growth
Source: Ecol Evol. 2023 Jul 30;13(8):e10295. doi: 10.1002/ece3.10295 (PMC10387745; doi:10.1002/ece3.10295)

(a)

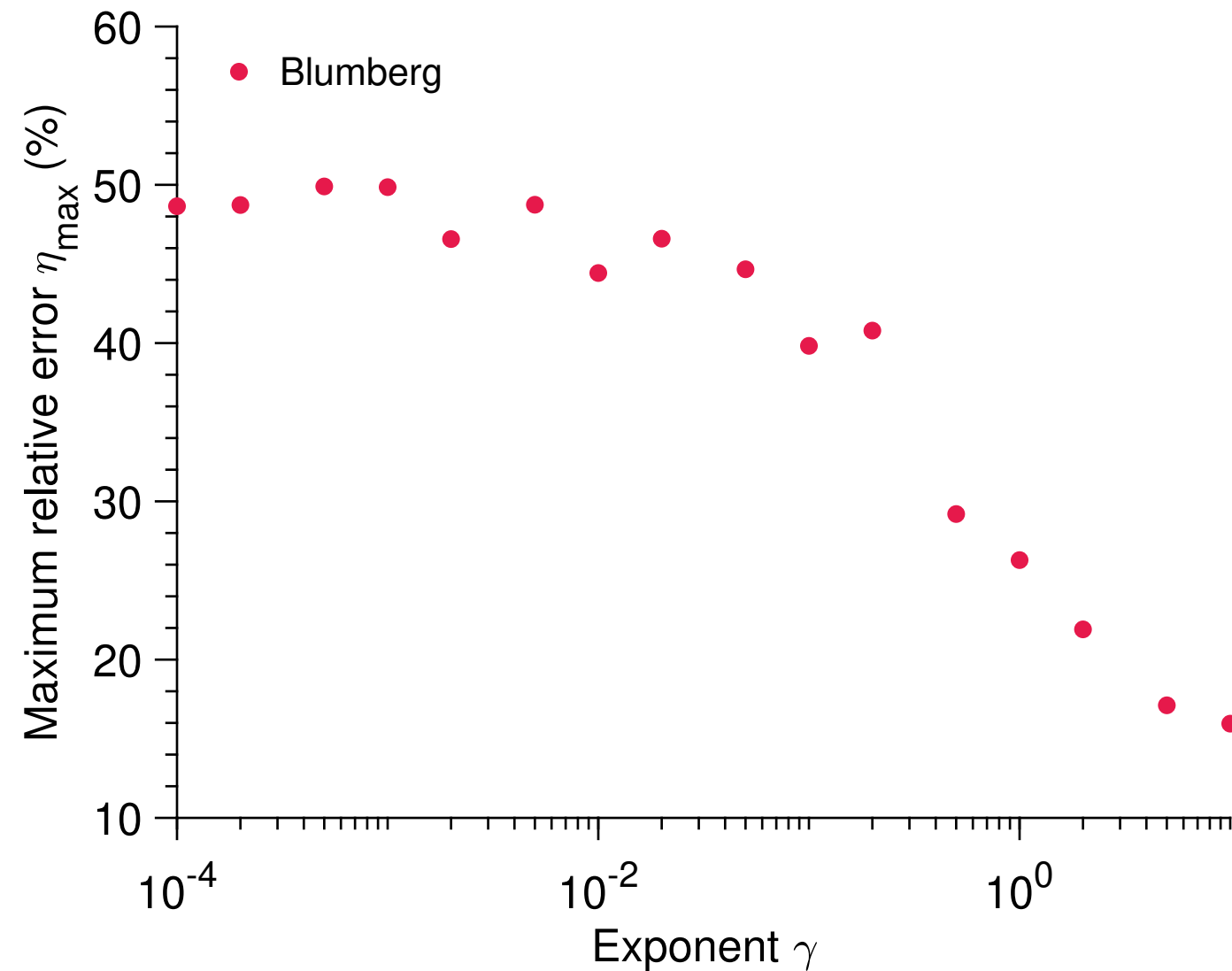

(b)

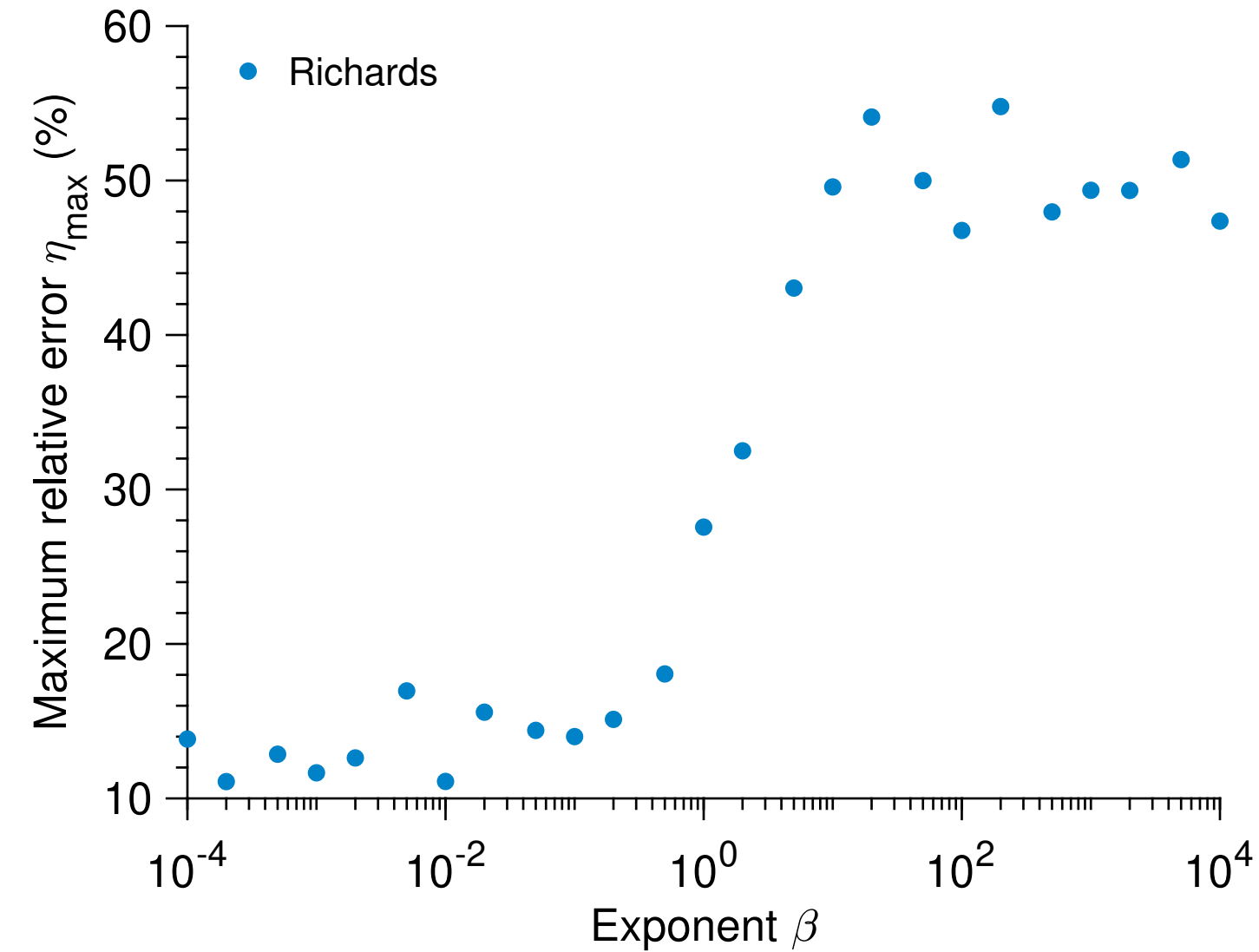

Supplement: Supplementary file 1 — Figure S1 [file ECE3-13-e10295-s002.pdf]

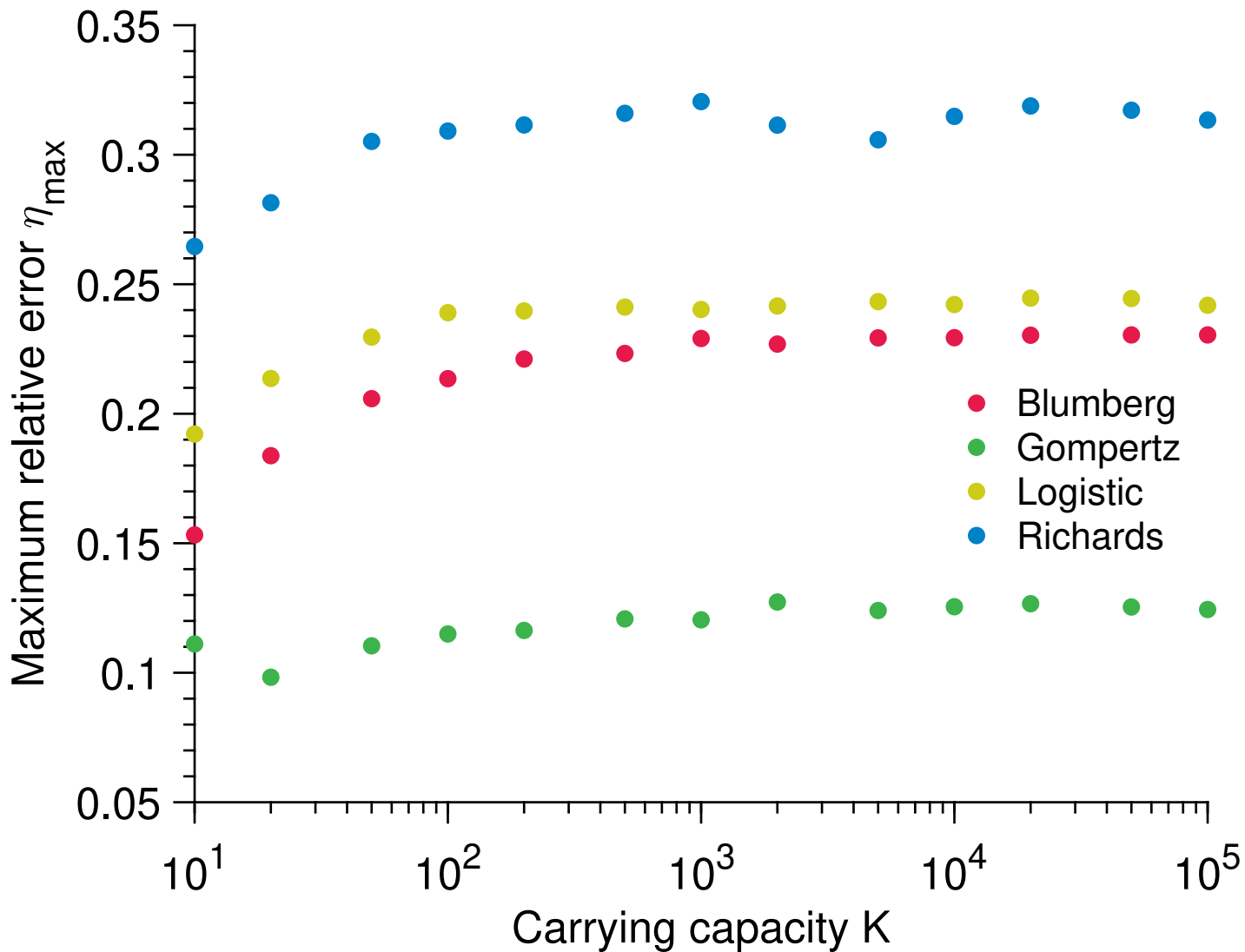

Supplement: Supplementary file 2 — Figure S2 [file ECE3-13-e10295-s003.pdf]

(a)

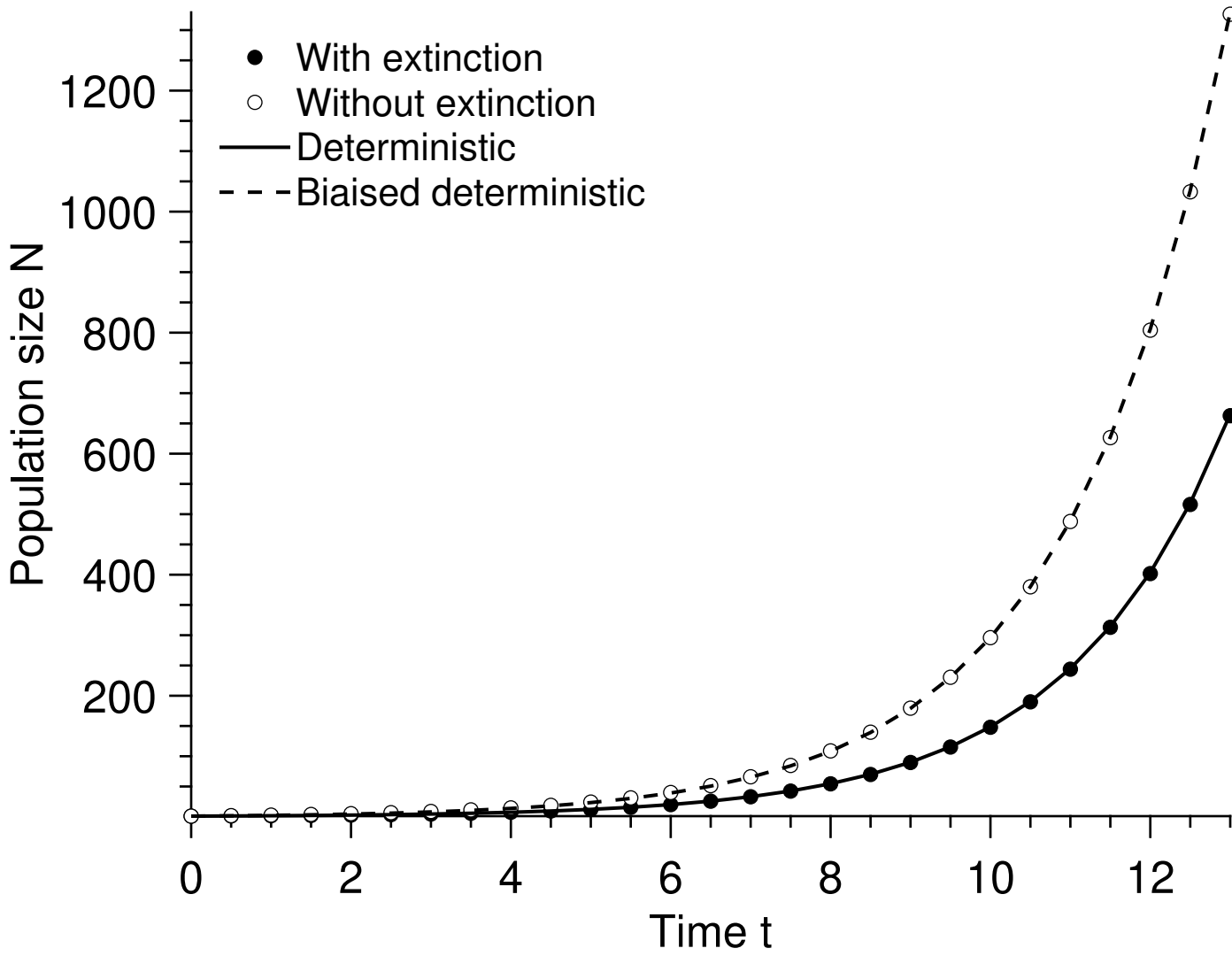

(b)

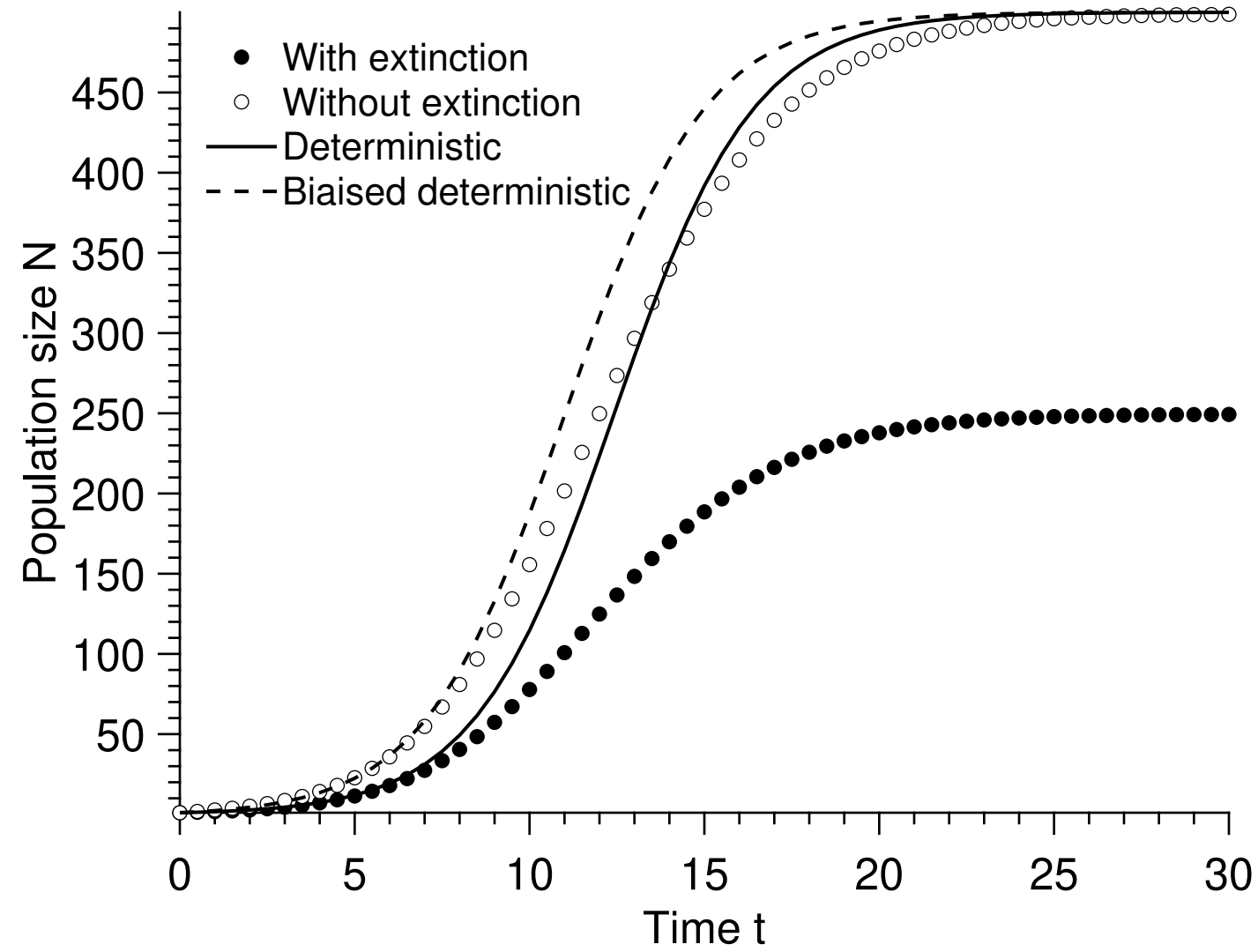

Supplement: Supplementary file 3 — Figure S3 [file ECE3-13-e10295-s001.pdf]
